# The monitoring of vancomycin: a systematic review and meta-analyses of area under the concentration-time curve-guided dosing and trough-guided dosing

**DOI:** 10.1186/s12879-021-05858-6

**Published:** 2021-02-06

**Authors:** Moeko Tsutsuura, Hiromu Moriyama, Nana Kojima, Yuki Mizukami, Sho Tashiro, Sumika Osa, Yuki Enoki, Kazuaki Taguchi, Kazutaka Oda, Satoshi Fujii, Yoshiko Takahashi, Yukihiro Hamada, Toshimi Kimura, Yoshio Takesue, Kazuaki Matsumoto

**Affiliations:** 1 Division of Pharmacodynamics, Keio University Faculty of Pharmacy, 1-5-30 Shibakoen, Minato-ku, Tokyo, 105-8512 Japan; 2 Department of Pharmacy, Kumamoto University Hospital, 1-1-1, Honjo, Chuo-ku, Kumamoto-shi, Kumamoto, 860-8556 Japan; 3 Department of Hospital Pharmacy, Sapporo Medical University Hospital, 16-291, South 1, West 16, Chuo-ku, Sapporo, Hokkaido 060-8543 Japan; 4 Department of Pharmacy, Hyogo College of Medicine, 1-1, Mukogawa-machi, Nishinomiya, 663-8501 Japan; 5 Department of Pharmacy, Tokyo Women’s Medical University Hospital, 8-1, Kawada-cho, Shinjuku-ku, Tokyo, 162-0054 Japan; 6 Department of Infection Control and Prevention, Hyogo College of Medicine, 1-1, Mukogawa-cho, Nishinomiya, 663-8501 Japan

**Keywords:** Vancomycin, Trough, AUC, Nephrotoxicity, Meta-analysis

## Abstract

**Background:**

This systematic review and meta-analysis explored the relationship between vancomycin (VCM) monitoring strategies and VCM effectiveness and safety.

**Methods:**

We conducted our analysis using the MEDLINE, Web of Sciences, and Cochrane Register of Controlled Trials electronic databases searched on August 9, 2020. We calculated odds ratios (ORs) and 95% confidence intervals (CIs).

**Results:**

Adult patients with methicillin-resistant *Staphylococcus aureus* (MRSA) bacteraemia with VCM trough concentrations ≥15 μg/mL had significantly lower treatment failure rates (OR 0.63, 95% CI 0.47–0.85). The incidence of acute kidney injury (AKI) increased with increased trough concentrations and was significantly higher for trough concentrations ≥20 μg/mL compared to those at 15–20 μg/mL (OR 2.39, 95% CI 1.78–3.20). Analysis of the target area under the curve/minimum inhibitory concentration ratios (AUC/MIC) showed significantly lower treatment failure rates for high AUC/MIC (cut-off 400 ± 15%) (OR 0.28, 95% CI 0.18–0.45). The safety analysis revealed that high AUC value (cut-off 600 ± 15%) significantly increased the risk of AKI (OR 2.10, 95% CI 1.13–3.89). Our meta-analysis of differences in monitoring strategies included four studies. The incidence of AKI tended to be lower in AUC-guided monitoring than in trough-guided monitoring (OR 0.54, 95% CI 0.28–1.01); however, it was not significant in the analysis of mortality.

**Conclusions:**

We identified VCM trough concentrations and AUC values that correlated with effectiveness and safety. Furthermore, compared to trough-guided monitoring, AUC-guided monitoring showed potential for decreasing nephrotoxicity.

**Supplementary Information:**

The online version contains supplementary material available at 10.1186/s12879-021-05858-6.

## Background

Vancomycin (VCM) is a broad-spectrum antibiotic that acts against Gram-positive bacteria, including methicillin-resistant *Staphylococcus aureus* (MRSA), and is used for the treatment of several infections [1, 2]. However, its use requires therapeutic drug monitoring (TDM) to ensure its therapeutic effectiveness and avoid nephrotoxicity.

A recent meta-analysis revealed that compared to low area under the curve/minimum inhibitor concentration ratios (AUC/MIC), high AUC/MIC ratios were associated with significantly lower mortality and treatment failure rates [3]. The practice guidelines for TDM of VCM recommended an AUC/MIC ratio of ≥400 to predict the clinical efficacy of VCM against MRSA (MIC ≤1 μg/mL) [4, 5]. However, Dalton et al. reported that the target AUC/MIC could not be calculated that related to the effectiveness and safety of VCM [6]. Therefore, the target AUC/MIC value, which is an indicator of effectiveness in MRSA infection therapy, is still controversial. On the other hand, in real-world clinical situations, trough concentrations are used as alternate indicators of AUC values, and in practice, target trough concentrations between 10 and 20 μg/mL are recommended to achieve an AUC/MIC ratio of ≥400 at MIC values of 0.5 and 1 μg/mL. Furthermore, in cases of serious infections such as bacteraemia, infective endocarditis, osteomyelitis, meningitis, and hospital-acquired and healthcare-associated pneumonia caused by MRSA, trough concentrations of 15–20 μg/mL are recommended to further improve patient outcomes [5, 7]. Thus, Tongsai et al. performed a meta-analysis to clarify the relationship between trough concentrations and effectiveness. They reported that no significant differences in mortality and treatment success rate between trough concentrations of ≥15 and < 15 μg/mL [8]. However, because AUC values increase as trough concentrations rise, it is unclear why or how this result was reached. Thus, a reanalysis of the relationship between trough concentrations and effectiveness is needed.

One of the adverse events associated with VCM use is acute kidney injury (AKI). Lodise et al. reported incidence rates for AKI of 21% for trough VCM concentrations of 10–15 μg/mL, 20% for 15–20 μg/mL, and 33% for ≥20 μg/mL [9]. Bellos et al. evaluated the risk of AKI at cutoff values of 10, 15, 20, and 25 μg/mL, and reported that the risk of AKI increased as the trough level increased [10]. These results indicate a clear relationship between AKI incidence and increased trough concentrations [9, 10]. A meta-analysis of AKI incidence indicated significantly higher incidence rates for trough concentrations ≥15 μg/mL compared to those for concentrations < 15 μg/mL [8, 10, 11]. In the clinical setting, dosage regimens based on the trough level is still used because it is a conventional method. As we mentioned above, the cutoff value for the effectiveness and safety of VCM is still under discussion, and it is important to clarify the optimal target value.

Recent evidence suggests that VCM-induced AKI correlates better with AUC values than with trough concentrations. For example, rat studies indicated that urine kidney injury molecule 1 (KIM-1) concentration was a useful indicator for the early detection of VCM-induced AKI [12] and that increases in urine KIM-1 concentration exhibited higher correlation coefficients with AUC values than with trough concentrations [13]. In their meta-analysis of clinical research, Aljefri et al. showed a significantly higher AKI incidence with a high AUC compared to that with a low AUC. They also reported that an AUC ≥650 μg × hr./mL is a risk indicator for AKI [14]. Furthermore, their meta-analysis judging the comparative usefulness of AUC-guided and trough-guided monitoring strategies for avoiding AKI showed that AUC-guided monitoring significantly reduced the AKI incidence compared to trough-guided monitoring [14]. However, this meta-analysis included only two papers; therefore, one would be hard-pressed to consider such a finding to be thoroughly demonstrated. Subsequent trials comparing AKI incidence rates associated with different VCM monitoring strategies have been reported [15, 16].

The present study performed a systematic review and meta-analysis to clarify the relationship between VCM trough concentrations or AUC values and its effectiveness and safety. Further, we studied whether trough-guided or AUC value-guided VCM monitoring strategies were more appropriate.

## Methods

### Search strategies

#### Search strategy for the evaluation of VCM target trough concentrations

We performed a literature search in the MEDLINE, Web of Science, and Cochrane Register of Controlled Trials electronic databases (August 92,020). Two of the four reviewers (MT, HM, NK, and YM) independently searched for literature using the following search terms: “vancomycin”, “trough”, and “monitoring”. The detailed search strategies are shown in Supplementary Table S1. Screening was conducted and duplicated articles were excluded.

#### Search strategy for the evaluation of VCM target AUC values

We performed a literature search in the MEDLINE, Web of Science, and Cochrane Register of Controlled Trials electronic databases (August 92,020). Two of the four reviewers (MT, HM, NK, and YM) independently searched for literature using the following search terms: “vancomycin”, “AUC”, or “area under the curve”. The detailed search strategies are shown in Supplementary Table S2. Screening was conducted and duplicated articles were excluded.

#### Search strategy for the evaluation of different monitoring strategies

We performed a literature search in the MEDLINE, Web of Science, and Cochrane Register of Controlled Trials electronic databases (August 92,020). Two of the four reviewers (MT, HM, NK, and YM) independently searched for literature using the following search terms: “vancomycin” or “monitoring”. The detailed search strategies are shown in Supplementary Table S3. Screening was conducted and duplicated articles were excluded.

### Study selection

#### Study selection for the evaluation of VCM target trough concentrations

A study was considered eligible for the evaluation of the VCM target trough concentrations it met the following criteria: trough levels were determined after the intravenous administration of VCM; more than two ranges of trough level were compared; data on the detailed outcomes regarding the effectiveness (clinical cure, treatment success or failure) or nephrotoxicity were available; and all subjects had MRSA bacteraemia only for analysis of the effectiveness. Studies that met the following exclusion criteria were excluded: questionnaire study, letter, case report, and review articles; non-adult patients or non-human subjects; and detailed results not available in English.

#### Study selection for the evaluation of the VCM target AUC values

A study was considered eligible for the evaluation of the VCM target trough concentrations it met the following criteria: AUC values were determined after the intravenous administration of VCM; more than two ranges of AUC values were compared; and available data on the detailed outcomes of the effectiveness (clinical response, treatment success or failure, mortality, or bacterial eradication) or nephrotoxicity. All subjects had MRSA bacteraemia only for the analysis of the effectiveness. Studies that met the following exclusion criteria were excluded: questionnaire study, letter, case report, and review articles; non-adult patients or non-human subjects; MICs determined by Etest, and detailed results not available in English.

#### Study selection for the evaluation of different monitoring strategies

A study was considered eligible for the evaluation of the VCM target trough concentrations it met the following criteria: a comparative study comparing AUC-guided monitoring and trough-guided monitoring of VCM; VCM was intravenously administrated; and availability of detailed outcomes regarding the effectiveness (clinical cure, treatment success or failure) or nephrotoxicity. Furthermore, studies that met the following exclusion criteria were excluded: questionnaire study, letter, case report, and review articles; non-adult patients or non-human subjects; the VCM dose was not adjusted based on AUC or trough; and detailed results not available in English.

Two of the four reviewers (MT, HM, NK, and YM) independently conducted the screening. When opinions differed, the screening results were determined based on discussions involving a third person (ST).

### Data extraction

Two of the four reviewers (MT, HM, NK, and YM) independently extracted data from the studies. When opinions differed, they were extracted through discussion with two additional people (ST and SO). The design, country, duration, age of patients, number of patients, target values (AUC and trough), causative bacteria, lesion, and rate of MRSA were extracted. The AUC definition method was also extracted in the evaluation of the AUC values.

### Outcomes analysis

#### Outcome analysis for the evaluation of VCM target trough concentrations

In the analysis of the relationship between trough levels of VCM and its effectiveness and nephrotoxicity, the primary outcomes (effectiveness and nephrotoxicity) were defined according to each study’s definition. Definitions of the nephrotoxicity criteria in each study are indicated in Table S4.

#### Outcome analysis for the evaluation of the VCM target AUCvalues

Unlike trough levels, the included studies showed considerable variation in the AUC/MIC ratio and AUC value cutoff in the comparison of clinical failure and adverse effects. Considering the differences in methodological and technical measurements, rounding within 15% of the AUC was considered appropriate for the meta-analysis. Therefore, the cut-off value of AUC/MIC ratios of 340–460 was defined as 400, and AUC values of 510–690 were defined as 600. The analysis of the relationship between the AUC values of VCM and its effectiveness and nephrotoxicity, the primary outcomes (effectiveness and nephrotoxicity) were defined as follows: effectiveness was defined as a clinical response, treatment failure, mortality, or bacterial eradication, while nephrotoxicity was defined as according to the 2009 VCM consensus guideline (a serum creatinine (SCr) increase of ≥0.5 mg/dL or ≥ 50% of the baseline SCr for ≥2 consecutive measurements) or AKIN stage 1 (SCr increase of ≥0.3 mg/dL or ≥ 1.5 times the baseline SCr).

#### Outcome analysis for the evaluation of different monitoring strategies

Our analysis of the differences in monitoring strategies defined effectiveness and safety as the primary outcome measures. Effectiveness was defined as mortality, while nephrotoxicity was defined according to the 2009 VCM consensus guideline or AKIN stage 1, as described above.

### Assessment of the risk of bias

Two of the four reviewers (MT, HM, NK, and YM) independently assessed the methodologic quality and risk of bias based on the Cochrane Collaboration (Risk Of Bias In Non-Randomized Studies of Interventions, ROBINS-I) [17]. Disagreements were resolved by discussion with a third person a resolution was reached.

### Analysis of the results and statistical analyses

We performed the meta-analysis using Review Manager for Windows (RevMan, Version 5.3, Copenhagen: The Nordic Cochrane Centre, The Cochrane Collaboration, 2014) and prepared forest plots. We calculated the odds ratios (ORs) and 95% confidence intervals (CIs) using a Mantel–Haenszel random-effects model. Statistical heterogeneity among studies was assessed using *I*^2^. *I*^2^ values of ≥50%, 25–50%, and ≤ 25% were regarded as strong, moderate, and no heterogeneity, respectively.

## Results

### Search results

**Fig. 1:**
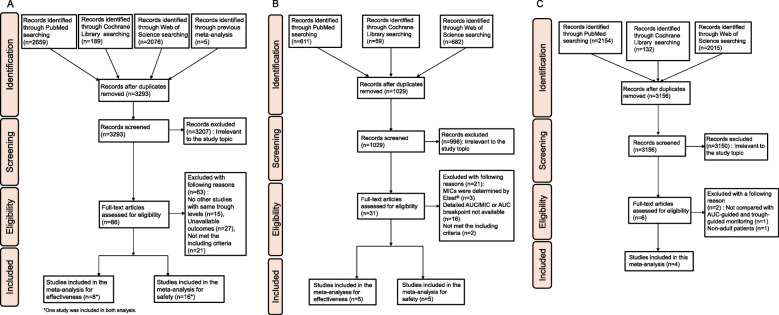
Flow chart of the selection process for studies. **a** Studies of trough-guided monitoring strategy associated with effectiveness and safety of VCM treatment. **b** Studies of AUC-guided monitoring strategy associated with effectiveness and safety of VCM treatment. **c** Studies of AUC-guided monitoring vs. trough-guided monitoring strategy associated with effectiveness and safety of VCM treatment

In database searching for VCM target trough concentrations evaluation, we obtained 3293 articles to be screened (Fig. 1a). Of these, 86 articles were further examined in detail, and finally, eight studies [18–25] were included in the meta-analysis for effectiveness evaluation, 16 studies [9, 20, 26–39] were included in the meta-analysis for safety evaluation, and one study was included in both analyses (Kullar 2011).

In database searching for VCM target AUC values evaluation, we obtained 1029 articles to be screened (Fig. 1b). Of these, 31 articles were further examined in detail, and finally, five studies [22, 23, 40–42] included in the meta-analysis for effectiveness evaluation, five studies [15, 43–46] were included in the meta-analysis for safety evaluation.

In database searching for the different monitoring strategies, we obtained 3156 articles to be screened (Fig. 1c). Of these, six articles were further examined in detail, and finally, four studies [15, 47–49] were included in the meta-analysis.

### Characteristics

The characteristics of the eight studies on effectiveness and the 16 studies on safety that were included in the meta-analysis of evaluating target trough concentrations are shown in Table 1. Of these, Song 2015, Obara 2016, Shime 2018, and de Almeida 2019 were prospective; the others were retrospective. The trough concentrations were measured at various times, including initial, mean, steady-state, and highest.

**Table 1:** Characteristics of included studies for target trough evaluation

Study	Design of study	Country	Duration of study	Age of patients	Percentage of MRSA and source	Definition of trough levels
Lodise 2009 [9]	Retrospective	America	2005–2006	≥18 Mean ± SD: 55.8 ± 18.1	MRSA infection (30%): Bloodstream, central nervous system, infective endocarditis, intra-abdominal, osteomyelitis, prophylaxis, respiratory tract, skin and soft tissue, urinary tract, unknown.	Highest The highest initial trough levels within 96 h of initiation of therapy
Hermsen 2010 [18]	Retrospective	America	2005–2007	≥19 Median (IQR): Trough < 15 μg/mL 59 (43–75) Trough ≥15 μg/mL 60 (44.5–70)	MRSA infection (100%): Pneumonia, endocarditis, osteomyelitis	Mean Trough levels calculated using the sum of each measured trough level multiplied by the number of days and divided by the total number of treatment days
Clemens 2011 [19]	Retrospective	America	2008–2009	≥18 Mean ± SD: 52.3 ± 16.3	MRSA bacteremia (100%): Skin or soft tissue/bone, intravascular catheter, respiratory, endocarditis, endovascular, abdominal, unknown.	Steady-state The first serum concentration collected ≤30 min before a scheduled dose after completing ≥24 h of vancomycin therapy
Kullar 2011 [20]	Retrospective	America	2005–2010	45–64 Median (IQR): Success 53 (45–64) Failure 54 (46–61)	MRSA bacteremia (100%): Skin/wound, catheter-related, endocarditis, pneumonia, bone and joint, deep abscess, multiple sites, other.	Steady-state Steady-state when available from clinical data. (e.g, immediately before the fourth dose)
Cano 2012 [26]	Retrospective	America	2006–2007	58.5 ± 17.2 Mean ± SD: 58.5 ± 17.2	Percentage of MRSA is not available: Hospital-acquired pneumonia, ventilator-associated pneumonia, health care–associated pneumonia	Highest Highest trough levels collected within 96 h of therapy
Horey 2012 [27]	Retrospective	America	2006–2008	≥18 Mean ± SD: 67.4 ± 12.5	Percentage of MRSA is not available: Empiric, skin and soft tissue, bone and joint, pneumonia, urinary tract infection, bacteremia/endocarditis, miscellaneous	Average The average levels were calculated by first multiplying each trough level by the number of days at that concentration; next, these values, from the total duration of therapy, were added. The sum was then divided by the total number of days of vancomycin exposure to produce a clinical picture of total exposure to vancomycin.
Prabaker 2012 [28]	Retrospective	America	2005–2007	Median 59 or 61 in each group	Percentage of MRSA is not available: Skin/soft tissue/bone infection, pneumonia, bacteremia, other.	Mean Trough levels drawn 30–60 min prior to the fourth dose, and again in 5–7 days or with any large change in renal function
Casapao 2013 [21]	Retrospective	America	2004–2012	≥18 Mean ± SD: 57 ± 15.4	MRSA bacteremia (100%): Infective endocarditis, pneumonia, intravenous catheter-related infection, bone and joint infection, skin and soft tissue infection, unknown.	Initial (No detail information is available.)
Fujii 2013 [29]	Retrospective	Japan	2011	> 18 Median (range), SD: 64 (21–88), 14.2	Percentage of MRSA is not available.	Highest Trough levels determined 3 days after the initiation of vancomycin therapy
Ley 2013 [30]	Retrospective	America	2006–2010	≥18 Mean ± SD: 50 ± 22.6	Percentage of MRSA is not available: Trauma.	Trough levels drawn 1 h prior to the subsequent dose
Barriere 2014 [31]	Retrospective	38 countries	2005–2007	≥18 Mean ± SD: 64.7 ± 16.2	MRSA pneumonia (78%): *S. aureus* nosocomial pneumonia, multilobar pneumonia, bacteremia.	Median (No detail information is available.)
Ghosh 2014 [22]	Retrospective	Australia	2006–2012	> 18 Median (range): 64.6 (22–95)	MRSA bacteremia (100%): Line-related bacteremia, bone and joint, skin and soft tissue infections, deep abscess, infective endocarditis, pneumonia, abdominal, non-endocarditis vascular, other, no identified focus.	Steady-state Trough levels obtained a minimum of 12 h after the last dose
Song 2015 [23]	Prospective	Korea	2010–2012	≥18 Median (IQR): 67 (53–75)	MRSA bacteremia (100%): Central venous catheter, bone and joint, skin and soft tissue, deep tissue abscess, lower respiratory tract, endovascular infection, urinary tract, intra-abdominal, unknown, high-risk source.	Initial (No detail information is available.)
Hammoud 2016 [33]	Retrospective	America	2011–2012	> 18 Mean: 56	MRSA infection (13%): Skin and soft tissue infection, pneumonia, osteomyelitis, pelvic/abdominal infection	Mean Mean levels calculated based on the theoretical number of days at various troughs for a specific patient
Hirano 2016 [34]	Retrospective	Japan	2007–2014	> 18 Mean ± SD: 68.2 ± 15.8	MRSA infection (100%): Respiratory, skin and soft tissue, bacteremia, Central nervous, Intra-abdominal, urinary tract, mediastinal, bone and joint.	Steady-state Trough levels defined as those determined after the fifth dose or on day 3 after the initiation of therapy
Obara 2016 [32]	Prospective	Brazil	2013–2014	> 18 Median (IQR): Trough 15–20 μg/mL 64.5 (52.3–79.5) Trough ≥20 μg/mL 55.5 (40–70.8)	Percentage of MRSA is not available.	Initial Initial levels obtained immediately before vancomycin fourth dose
Chuma 2018 [35]	Retrospective	Japan	2005–2015	≥18 Median (IQR): 67 (55–75)	MRSA infection (34%): Abdominal, blood stream catheter related, endocarditis, meningitis, soft tissue, pulmonary, urinary.	Initial Initial trough levels measured within 4 days after the beginning of administration
Fu 2018 [24]	Retrospective	Taiwan	2013–2016	≥20 Mean ± SD: 69 ± 14.8	MRSA bacteremia (100%): Bone and joint, catheter-related, endocarditis, pneumonia, surgical wound or skin and soft tissue, unknown.	Mean Pre-dialysis trough levels
Huang 2018 [36]	Retrospective	China	2007–2014	≥80 Mean ± SD: 85 ± 3.9	MRSA infection (24%)	Trough levels obtained within 72 h of commencing therapy, after administering a minimum of three doses
Mogle 2018 [25]	Retrospective	America	2016–2018	≥18 Mean ± SD: 50 ± 17.6	MRSA bacteremia (100%): Skin and soft tissue, catheter related/endovascular, bone and joint, urinary tract, pneumonia, presence of endocarditis, unknown.	Steady-state consecutive steady-state post-distributional serum concentrations obtained within 96 h of therapy
Park 2018 [37]	Retrospective	Korea	2013	≥18 Median (IQR): 58 (45–59)	Percentage of MRSA is not available: Pneumonia, sepsis/Septic shock, skin/skin structure infection, bacteremia, other.	Mean Trough levels measured in blood samples collected just prior to administration of the next dose
Shime 2018 [38]	Prospective	Japan	2014–2015	60–78 Median (IQR): 71 (60–78)	MRSA infection (100%): Bacteremia, lung skin and soft tissue, bone and joint, other.	Highest (No detail information is available.)
de Almeida 2019 [39]	Prospective	Brazil	2017–2018	≥18 Median (IQR): 55.9 (40.6–66.8)	MRSA infection (6.1%): Skin and soft tissue, surgical site, pulmonary, bone, catheter, central nervous system, kidney, others, undetermined.	Steady-state Trough levels measured at the third (after the fourth or fifth dose, corresponding to the steady-state)

*N/A* not available

The characteristics of the five studies on effectiveness and five studies on safety that were included in our meta-analysis evaluating target AUC values are shown in Tables 2 and 3, respectively. Three studies (Song 2015, Meng 2019, and Lodies 2020) were prospective studies; others were retrospective.

**Table 2:** Characteristics of the included studies for target AUC/MIC based on effectiveness

Study	Design of study	Country	Duration of study	Age of patients	Patient’s condition	Definition of AUC values	Target AUC/MIC breakpoint
Zelenitsky 2013 [40]	Retrospective	Canada, America, Saudi Arabia	1996–2005	≥18 Mean ± SD: 55.9 ± 16.7	MRSA-associated septic shock	Values calculated (i) within the first 72 h of therapy based on the measured and extrapolated serum levels, and (ii) at steady-state using the daily dose divided by the population pharmacokinetic model derived vancomycin clearance	≥ 451
Ghosh 2014 [22]	Retrospective	Australia	2006–2012	> 18 Median (range): 64.6 (22–95)	MRSA bacteremia	D/(CLcr × 0.79) + 15.4] × 0.06	≥ 398
Jung 2014 [41]	Retrospective	Korea	2009–2012	≥18 Median (IQR): 69 (34–93)	MRSA bacteremia	Values estimated fitting vancomycin serum levels to a two-compartment volume clearance model using the maximum a posteriori probability Bayesian approach	≥ 398.5
Song 2015 [23]	Prospective	Korea	2005–2007	≥18 Median (IQR): 67 (53–75)	MRSA bacteremia	The total vancomycin dose in milligrams for 24 h over the vancomycin clearance	≥ 392.7
Makmor-bakry 2019 [42]	Retrospective	Malaysia	N/A	≥18 Mean ± SD: 59.2 ± 14.5	MRSA bacteremia	Values estimated from the trough level and published vancomycin population PK values	≥ 400

*D* vancomycin dosage in mg/24 h, *CLcr* estimated creatinine clearance, *N/A* not available

**Table 3:** Characteristics of the included studies for target AUC based on nephrotoxicity

Study	Design of study	Country	Duration of study	Age of patients	Patient’s condition	Definition of AUC values	Target AUC breakpoint
Chavada 2017 [43]	Retrospective	Australia	2006–2012	> 18% of patient age ≥ 70: AKI 50.0% Non-AKI 41.1%	MRSA bacteremia	Values estimated by the maximum a posteriori Bayesian estimation, using a priori pharmacokinetic parameters of a previous population pharmacokinetic model	≥ 563
Zasowski 2018 [44]	Retrospective	America	2014–2015	> 18 Mean ± SD: 61.7 ± 16.8	Confirmed or suspected bacteremiaor pneumonia	Values estimated via the maximum a posteriori probability Bayesian function using a previously published 2-compartment population pharmacokinetic model as the Bayesian prior	≥ 683
Meng 2019 [15]	Prospective	America	2018	≥18 Median ± SD (IQR): AKI 51 ± 19 (37–62) Non-AKI 63 ± 17 (50–69)	Pulmonary, skin and soft tissue infection, osteoarticular, febrile neutropenia, abdominal, pelvic, intrathoracic, bacteremia, central nervous system, endocarditis, cardiovascular implantable, electronic device infections, vascular graft	Values obtained by a Stanford hospital–specific spreadsheet calculator with prebuilt pharmacokinetic equations using Microsoft Excel (http://med.stanford.edu/bugsanddrugs.html)	≥ 600
Brunetti 2020 [45]	Retrospective	America	2011–2018	≥18 Mean ± SD: 57 ± 16.4	N/A	Values estimated by DoseMe software, which uses a Bayesian approach	> 600
Lodise 2020 [46]	Prospective	America	2014–2015	≥18 Mean ± SD: 60.7 ± 17.3	MRSA bloodstream infection	Values estimated post hoc using the maximal a posteriori probability procedure	≥ 550

*N/A* not available

The characteristics of the four studies included in our meta-analysis of differences in monitoring strategies are shown in Table 4. The target AUC values were as follow: Finch 2017: 400–600 μg × hr./mL, Neely 2018, Meng 2019: 400–800 μg × hr./mL, and Oda 2020: > 400 μg × hr./mL. Target trough concentrations were as follows: Finch 2017 and Oda 2020, 15–20 μg/mL and Neely 2018 and Meng 2019, 10–20 μg/mL.

**Table 4:** Characteristics of the included studies for AUC and trough-guided monitoring

Study	Design of study	Country	Duration of study	Age of patients	Number of patients		Target AUC (mg*hr./L)	Target trough (mg/L)	Rate (%) of MRSA
					AUC-guided	Trough-guided			
Finch 2017 [47]	Retrospective, quasi-experimental study	America	2014–2015	≥ 18 Mean ± SD: 59.1 ± 16.9	734	546	400–600	15–20	N/A
Neely 2018 [48]	3-year, prospective, serial cohort study	America	2012–2016	≥ 18 Mean (range): 48.7 (18–93)	177	75	400–800	10–20	10
Meng 2019 [15]	Prospective observational quality assurance study	America	2017–2018	≥ 18 Median ± SD (IQR): Trough-guided 58 ± 17 (46–67) AUC-guided 62 ± 17 (46–68)	117	179	400–800	10–20	9
Oda 2020 [49]	Single-centered retrospective study	Japan	2016–2020	≥ 19 Median (range): Trough-guided 68.5 (19–84) AUC-guided 64.0 (19–87)	22	52	400–600	15–20	36

*N/A* not available

### Assessment of the risks of bias

**Fig. 2:**
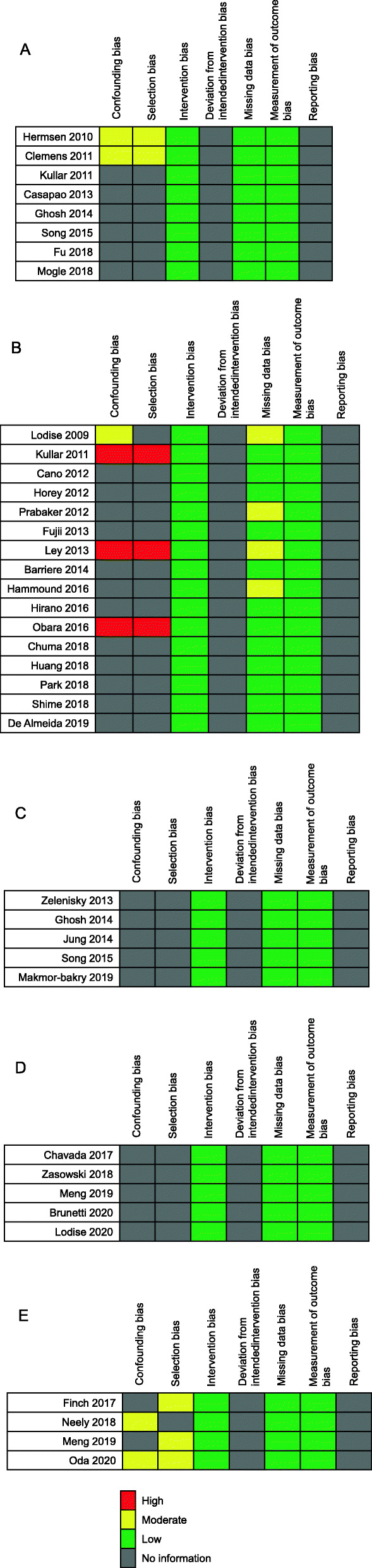
Methodological quality summary for each included study. The studies included in the evaluation of target trough concentration for **a** effectiveness and **b** safety. The studies included in the evaluation of **c** target AUC/MIC for effectiveness and **d** AUC for safety. **e** The studies included in the evaluation of effectiveness and safety associated with VCM monitoring strategy

The results of the assessment of the risk of bias are presented in Fig. 2. Three studies (Kullar 2011, Ley 2013, and Obara 2016) showed high risks of confounding and selection biases. No problems in intervention bias were identified and few problems were identified regarding missing data and measurement of outcome biases. No information was available for deviation from the intended intervention and reporting biases.

### Outcome analysis for the association of VCM target trough concentrations with effectiveness in adult patients

**Fig. 3:**
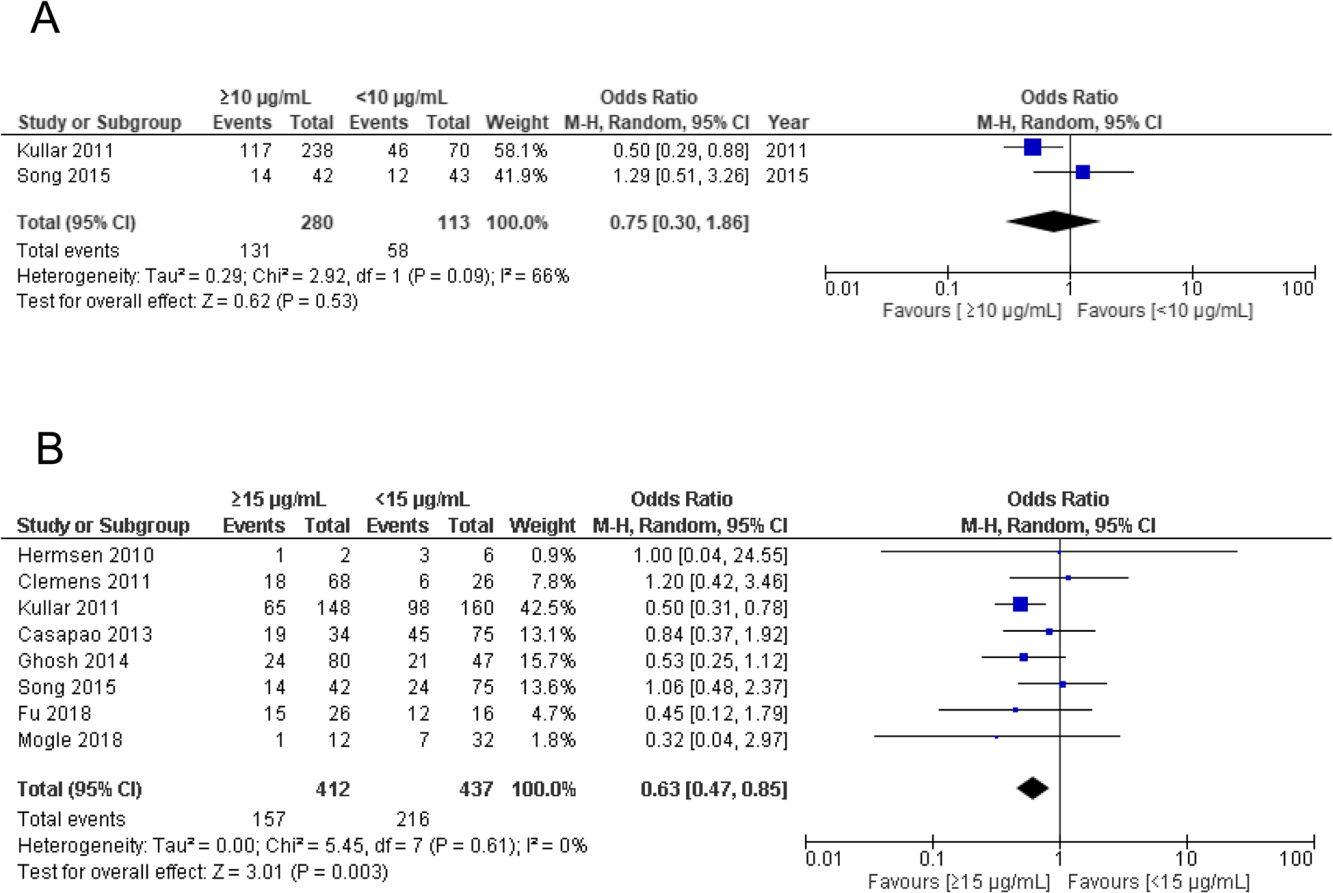
Forest plot of the treatment failure associated with VCM trough concentration. The vertical line indicates no significant difference between the groups compared. Diamond shapes and horizontal lines represent ORs and 95% CIs, respectively. Squares indicate point estimates, and the size of each square indicates the weight of each study included in this meta-analysis. **a** ≥10 μg/mL vs. < 10 μg/mL. **b** ≥15 μg/mL vs. < 15 μg/mL

The VCM trough concentrations were divided into two groups, ≥10 μg/mL and < 10 μg/mL or ≥ 15 μg/mL and < 15 μg/mL, and a meta-analysis of treatment failure was performed. Both studies included in Fig. 3a were on MRSA bacteraemia. No significant difference in treatment failure was observed for VCM trough concentrations ≥10 μg/mL vs. < 10 μg/mL (OR 0.75, 95% CI 0.30–1.86, *p* = 0.53) (Fig.3a). However, trough concentrations ≥15 μg/mL had significantly lower treatment failure rates than those of < 15 μg/mL in patients with MRSA bacteraemia (OR 0.63, 95% CI 0.47–0.85, *p* = 0.003) (Fig. 3b). We also performed this analysis in patients with MRSA infection, which was not restricted to bacteraemia, and found no significant differences between ≥15 μg/mL and < 15 μg/mL (OR 0.85, 95% CI 0.58–1.25) (Supplementary Fig. S1).

### Outcome analysis for the association between VCM target trough concentrations and safety

**Fig. 4:**
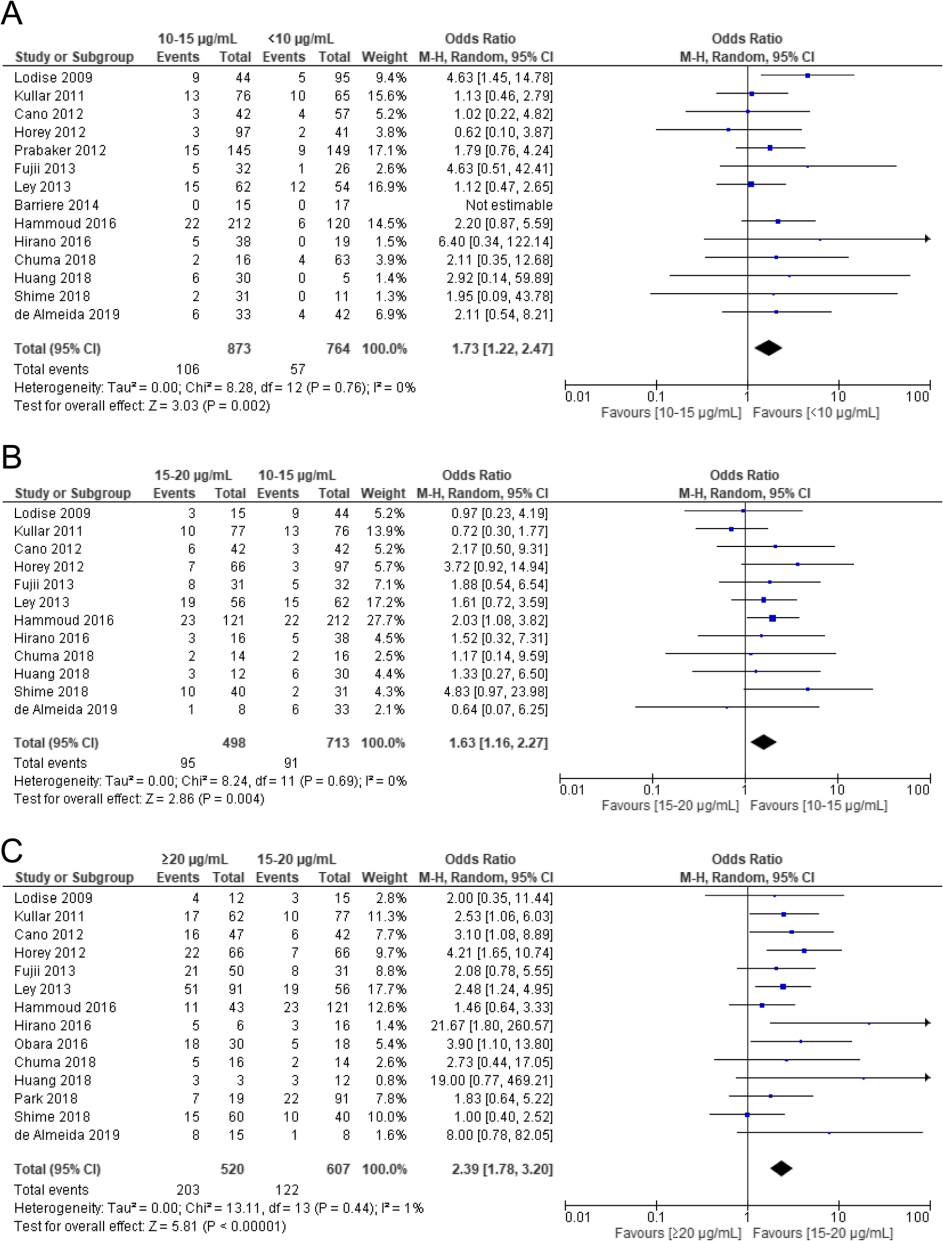
Forest plot of the risk of nephrotoxicity associated with VCM trough concentration. The vertical line indicates no significant difference between the groups compared. Diamond shapes and horizontal lines represent ORs and 95% CIs, respectively. Squares indicate point estimates, and the size of each square indicates the weight of each study included in this meta-analysis. **a** 10–15 μg/mL vs. < 10 μg/mL. **b** 15–20 μg/mL vs. 10–15 μg/mL. **c** > 20 μg/mL vs. 15–20 μg/mL

Trough concentrations were granularly divided into several categories before performing the meta-analysis. As shown in Fig. 4, the AKI incidence rates were significantly higher for (1) trough concentrations of 10–15 μg/mL compared to those < 10 μg/mL (OR 1.73, 95% CI 1.22–2.47, *p* = 0.002), (2) trough concentrations of 15–20 μg/mL compared to those of 10–15 μg/mL (OR 1.63, 95% CI 1.16–2.27, *p* = 0.004), (3) trough concentrations > 20 μg/mL compared to those 15–20 μg/mL (OR 2.39, 95% CI 1.78–3.20, *p* < 0.00001). The AKI incidence rates increased higher as VCM trough concentrations increased, with ORs markedly increasing at concentrations ≥20 μg/mL (Fig. 4c).

### Outcome analysis for the association between VCM target AUC/MIC ratios and AUC values with effectiveness and safety

**Fig. 5:**
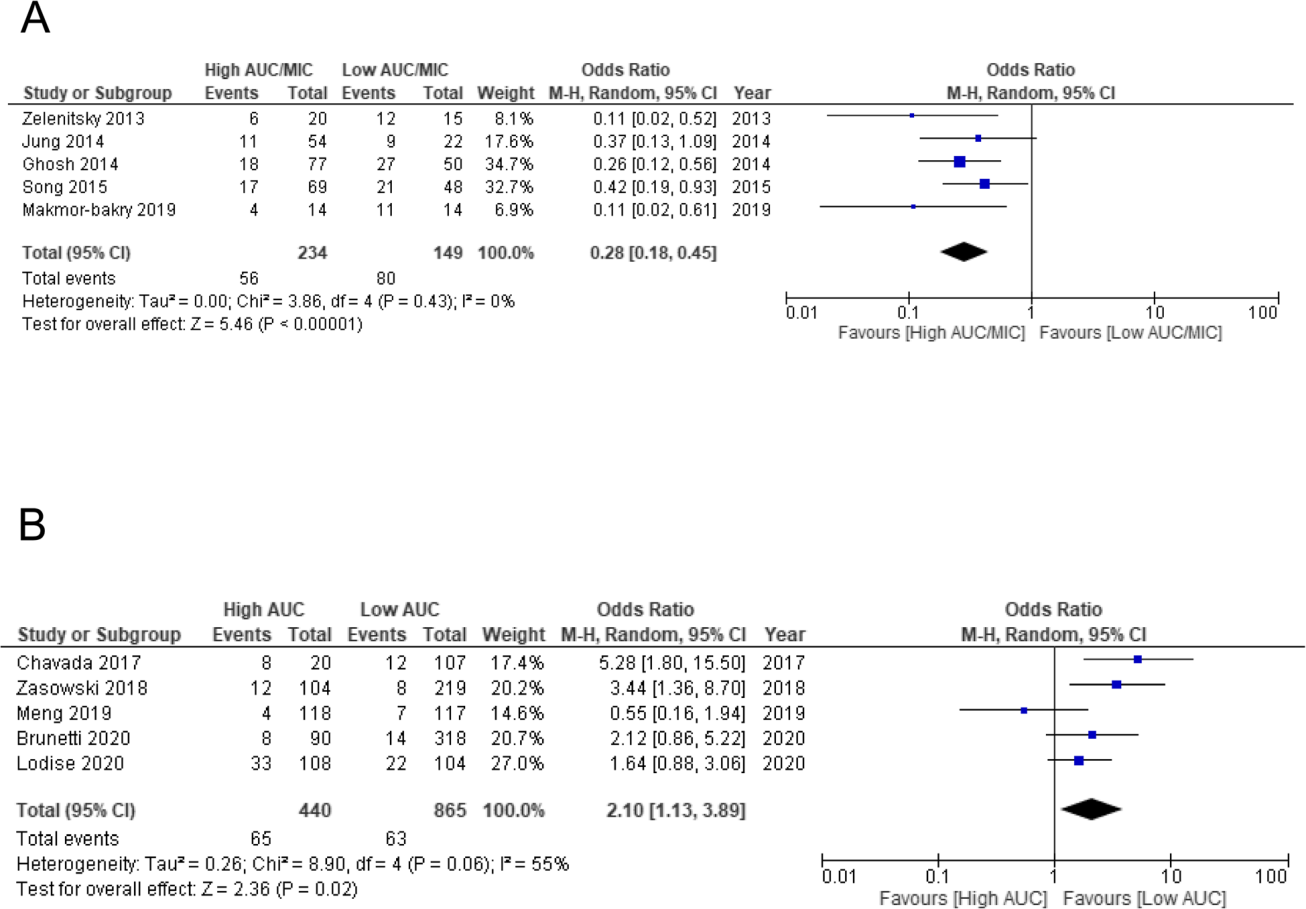
Forest plot of treatment failure and risk of nephrotoxicity associated with VCM AUC/MIC ratio and AUC value. The vertical line indicates no significant difference between the groups compared Diamond shapes and horizontal lines represent ORs and 95% CIs, respectively. Squares indicate point estimates, and the size of each square indicates the weight of each study included in this meta-analysis. **a** The OR of treatment failure associated with AUC/MIC ratios restricted with 400 ± 15% (392.7–451). **b** The OR of risk of nephrotoxicity associated with AUC values restricted with 600 ± 15% (550–683)

As analysis conducted using an AUC/MIC cutoff of 400 (400 ± 15%, 392.7–451) as an indicator of effectiveness showed that compared to low AUC/MIC ratios, high AUC/MIC ratios had significantly lower treatment failure rates (OR 0.28, 95% CI 0.18–0.45, *p* < 0.0001) (Fig. 5a). Based on the results of the analysis conducted using an AUC cut-off of 600 (600 ± 15%, 550–683) as an indicator of safety, the VCM-induced AKI incidence rates were significantly higher for high AUC values than for low AUC values (OR 2.10, 95% CI 1.13–3.89, *p* = 0.02) (Fig. 5b).

### Outcome analysis of the differences between monitoring strategies

**Fig. 6:**
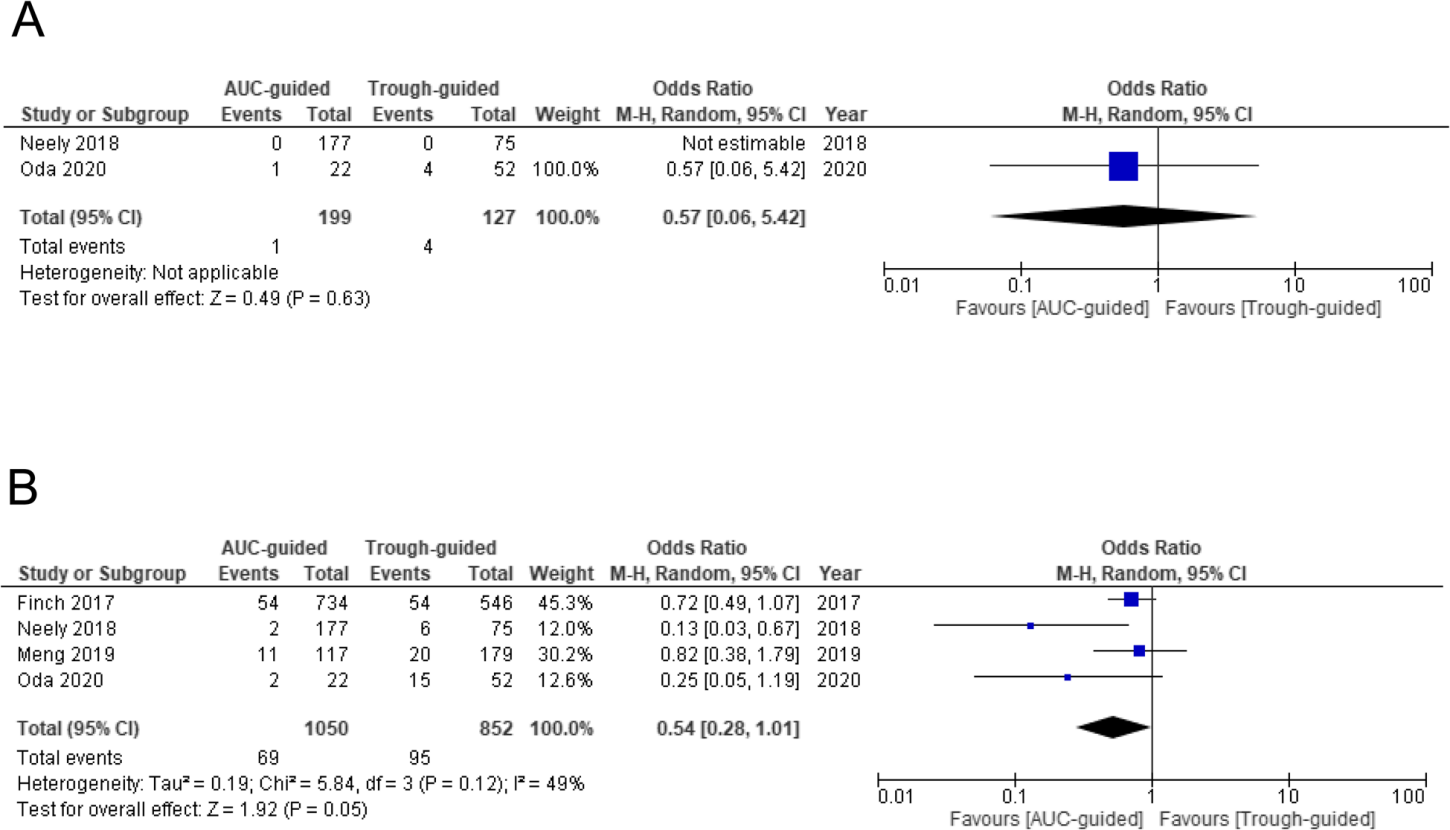
Forest plot of the effectiveness and risk of nephrotoxicity associated with VCM monitoring. The vertical line indicates no significant difference between the groups compared Diamond shapes and horizontal lines represent ORs and 95% CIs, respectively. Squares indicate point estimates, and the size of each square indicates the weight of each study included in this meta-analysis. **a** The OR of mortality associated with different monitoring strategies. **b** The OR of risk of nephrotoxicity associated with the difference in monitoring strategy

The mortality rates did not differ significantly between AUC-guided monitoring and trough-guided monitoring (OR 0.57, 95% CI 0.06–5.42) (Fig. 6a). While the incidence of AKI associated with VCM tended to be lower for AUC-guided monitoring than for trough-guided monitoring, the difference was not significant (OR 0.54, 95% CI 0.28–1.01, *p* = 0.05) (Fig. 6b).

## Discussion

We performed a meta-analysis to evaluate the effectiveness and safety of VCM trough concentrations. We observed a significantly lower treatment failure rate among bacteraemia patients with trough concentrations ≥15 μg/mL. However, we observed no significant difference in patients with MRSA infection. This may be because the subject population studied in the latter case including a large number of MRSA pneumonia patients. Even when MRSA is detected in a patient’s sputum, it is often not the causative agent of the infection in question, but rather a colonising species, making the diagnosis of MRSA pneumonia extremely difficult [50–53]. However, when bacteraemia is concerned, the causative agent can be definitively identified as MRSA through blood culture. We found that trough concentrations of ≥15 mg/L were used for the treatment of MRSA bacteraemia.

We also performed a meta-analysis to explore the relationship between trough concentrations and AKI incidence through granularly defined trough concentration categories. The AKI incidence rates significantly increased as trough concentrations increased. Particularly, when trough concentrations were ≥ 20 μg/mL, the odds ratio of AKI markedly increased. Several reports have explored the relationship between VCM-induced AKI incidence and trough concentrations [8, 10, 33, 54]. AKI incidence rates reportedly increase with trough concentrations ≥15 μg/mL and further increased for trough concentrations ≥20 μg/mL [8, 10, 54]. Thus, we believe that VCM trough concentrations should be kept below 20 μg/mL at all times and minimised wherever possible.

The AUC value is the best indicator of VCM effectiveness and safety. To define the target AUC values for effectiveness, we performed analyses based on an AUC/MIC cutoff value of 400 ± 15%. We observed that high AUC/MIC ratios were significantly superior to low AUC/MIC ratios. Similar to that of effectiveness, we also performed an analysis based on an AUC cutoff values of 600 ± 15% to define the target AUC values for safety. We observed that high AUC values significantly increased the AKI incidence rates. Consistent with our results and previous reports [4, 55], the recommended target AUC value threshold for avoiding VCM-induced AKI is approximately 600 μg × h/mL. While, trough concentrations are used primarily as alternate indicators of AUC values, recent reports suggest that the measurement of trough concentrations alone is not sufficient for the proper evaluation of AUC values [56–59]. Neely et al. showed that a correlation coefficient (r^2^) of 0.94 between AUC values calculated with VCM concentrations measured from blood collected at five or six points and AUC values calculated using peak and trough concentrations. The correlation coefficient (r^2^) between the former AUC values and those calculated using only trough concentrations was 0.70. Other reports also indicate that at least two points of measurement of peak and trough concentrations are needed to accurately calculate AUC levels. The present study incorporated two new reports to those used by Aljefri et al. in their analysis [14] and carried out a meta-analysis of the relationship between the incidence of kidney injury and AUC-guided vs. trough-guided monitoring. We found that AUC-guided monitoring was associated with lower incidence rates of kidney injury. However, the mortality rates did not differ significantly between AUC-guided monitoring and trough-guided monitoring. The target AUC values and trough concentrations in each study incorporated into this meta-analysis differed. Dalton et al. concluded that it was difficult to calculate the optimal target AUC/MIC as the AUC estimation method and study background varied among the studies [6]. In the future, a comparative trial of AUC-guided vs. trough-guided monitoring with appropriately defined target AUC values and trough concentrations is needed to determine if AUC-guided monitoring lowers the risk of mortality and AKI.

This study has subject to several limitations. First, most of the reports incorporated in our analyses were observational studies. The design of these studies may result in allocation bias, selection bias, and various types of other confounding factors in our results. Further, publication bias, in particular, is quite likely; that is, the idea that papers that demonstrate an effect of monitoring strategy differences on the primary outcome (AKI incidence) are preferentially selected and published. Second, the trials included in this study used several different definitions of AKI. Third, the detailed MICs of VCM were not available in the analysis of the effectiveness of VCM target trough concentrations. Therefore, we could not perform a subgroup analysis by MIC. Fourth, the methods used for the calculation of AUC values varied considerably among papers. Thus, to address these issues, future research efforts should involve large-scale prospective randomised clinical trials, which will enable further high-quality meta-analyses.

## Conclusion

This systematic review and meta-analysis identified trough concentrations and AUC values of VCM associated with its effectiveness and safety. Furthermore, compared to trough-guided monitoring, AUC-guided monitoring showed higher potential to reduce the incidence of VCM-induced AKI. Further high-quality trials exploring monitoring strategies for VCM use and the safety and effectiveness of VCM are needed to expand the research horizons in this area.

## Supplementary Information

**Additional file 1 **: **Table S1.** Search strategy for the evaluation of VCM target trough concentrations. **Table S2.** Search strategy for the evaluation of VCM target AUC values. **Table S3.** Search strategy for the evaluation of different monitoring strategies. **Table S4.** Definitions of outcome criteria in included studies.

**Additional file 2 **: **Fig. S1.** Forest plot of the treatment failure associated with VCM trough concentra-tion in patients with all MRSA infection. The vertical line indicates no significant difference between the groups compared. Dia-mond shapes and horizontal lines represent ORs and 95% CIs, respectively. Squares in-dicate point estimates, and the size of each square indicates the weight of each study in-cluded in this meta-analysis. VCM trough concentrations were divided into ≥15 μg/mL and < 15 μg/mL.

## Data Availability

The datasets used and/or analysed during the current study are available from the corresponding author upon reasonable request.

## Abbreviations

VCM: Vancomycin
MRSA: Methicillin-resistant *Staphylococcus aureus*
TDM: Therapeutic drug monitoring
AUC: Area under the curve
MIC: Minimum inhibitor concentration
AKI: Acute kidney injury
OR: Odds ratio
CI: Confidence interval
