# Supplementary material for: The monitoring of vancomycin: a systematic review and meta-analyses of area under the concentration-time curve-guided dosing and trough-guided dosing
Source: BMC Infect Dis. 2021 Feb 6;21:153. doi: 10.1186/s12879-021-05858-6 (PMC7866743; doi:10.1186/s12879-021-05858-6)
Supplement: Supplementary file 1 — Additional file 1 : Table S1. Search strategy for the evaluation of VCM target trough concentrations. Table S2. Search strategy for the evaluation of VCM target AUC values. Table S3. Search strategy for the evaluation of different monitoring strategies. Table S4. Definitions of outcome criteria in included studies. [file 12879_2021_5858_MOESM1_ESM.docx]

Table S1 Search strategy for VCM target trough concentrations evaluation

| Data base: MEDLINE | | |
| --- | --- | --- |
| # | Search term | Number of study |
| 1 | Vancomycin | 31117 |
| 2 | Monitoring | 1021123 |
| 3 | Trough | 19605 |
| 4 | #2 or #3 | 1036319 |
| 5 | #1 and #4 | 2659 |
|  |  |  |
| Data base: Cochrane Register of Controlled Trials | | |
| # | Search term | Number of study |
| 1 | Vancomycin | 2069 |
| 2 | Monitoring | 96006 |
| 3 | Trough | 6512 |
| 4 | #2 or #3 | 101459 |
| 5 | #1 and #4 | 189 |
|  |  |  |
| Data base: Web of Science | | |
| # | Search term | Number of study |
| 1 | Vancomycin | 34901 |
| 2 | Monitoring | 1474294 |
| 3 | Trough | 50417 |
| 4 | #2 or #3 | 1519879 |
| 5 | #1 and #4 | 2076 |

Table S2 Search strategy for VCM target AUC values evaluation

| Data base: MEDLINE | | |
| --- | --- | --- |
| # | Search term | Number of study |
| 1 | Vancomycin | 31117 |
| 2 | AUC | 75639 |
| 3 | Area under the curve | 125857 |
| 4 | #2 or #3 | 153539 |
| 5 | #1 and #4 | 611 |
|  |  |  |
| Data base: Cochrane Register of Controlled Trials | | |
| # | Search term | Number of study |
| 1 | Vancomycin | 2069 |
| 2 | AUC | 19353 |
| 3 | Area under the curve | 29793 |
| 4 | #2 or #3 | 36431 |
| 5 | #1 and #4 | 59 |
|  |  |  |
| Data base: Web of Science | | |
| # | Search term | Number of study |
| 1 | Vancomycin | 34943 |
| 2 | AUC | 79251 |
| 3 | Area under the curve | 123873 |
| 4 | #2 or #3 | 156503 |
| 5 | #1 and #4 | 682 |

Table S3 Search strategy for evaluation of different monitoring strategies

| Data base: MEDLINE | | |
| --- | --- | --- |
| # | Search term | Number of study |
| 1 | Vancomycin | 31117 |
| 2 | Monitoring | 1021123 |
| 3 | #1 and #2 | 2154 |
|  |  |  |
| Data base: Cochrane Register of Controlled Trials | | |
| # | Search term | Number of study |
| 1 | Vancomycin | 2069 |
| 2 | Monitoring | 96006 |
| 3 | #1 and #2 | 132 |
|  |  |  |
| Data base: Web of Science | | |
| # | Search term | Number of study |
| 1 | Vancomycin | 34943 |
| 2 | Monitoring | 1474294 |
| 3 | #1 and #2 | 2015 |

Table S4 Definitions of outcome criteria in included studies.

| study | Definition |
| --- | --- |
| Lodise 2009 | serum creatinine (SCr) increase of ≥ 0.5 mg/dL or ≥ 50% from the baseline |
| Hermsen 2010 | failure: worse, develop new symptom, require a new drug, persistence of MRSA for > 90 days, or death |
| Clemens 2011 | treatment failure: death due to MRSA within 30 days, persistence of MRSA bacteremia for > 10 days, or recurrence of MRSA bacteremia within 30 days |
| Kullar 2011 | serum creatinine (SCr) increase of ≥ 0.5 mg/dL or ≥ 50% from the baseline SCr for ≥ 2 consecutive measurements  vancomycin failure: death in 30 days, symptoms persist after treatment, or persistence of bacteremia for > 7days |
| Cano 2012 | serum creatinine (SCr) increase of ≥ 0.5 mg/dL or ≥ 50% from the baseline SCr 72 hr after drug administration |
| Horey 2012 | serum creatinine (SCr) increase of ≥ 0.5 mg/dL for 2 days consecutive measurements or ≥ 50% from the baseline SCr |
| Prabaker 2012 | serum creatinine (SCr) increase of ≥ 0.5 mg/dL or ≥ 50% from the baseline SCr for ≥ 2 consecutive measurements |
| Casapao 2013 | Failure of vancomycin treatment: persistence of bacteremia for > 7 days, develop new symptoms, death within 30 days from initial positive blood culture, blood culture positive of MRSA within 60 days after antimicrobial treatment |
| Ley 2013 | serum creatinine (SCr) increase of ≥ 0.5 mg/dL or ≥ 50% from the baseline |
| Barriere 2014 | serum creatinine (SCr) increase of ≥ 1.5 mg/dL or ≥ 150% from the baseline |
| Ghosh 2014 | treatment failure: death within 30 days, persistence of bacteremia, persistence of MRSA symptoms from initial treatment of VCM |
| Song 2015 | treatment failure: all causes mortality, persistence of bacteremia, or recurrence of MRSA bacteremia after end of antimicrobial therapy |
| Hammoud 2016 | serum creatinine (SCr) increase of ≥ 0.5 mg/dL or ≥ 50% from the baseline SCr for ≥ 2 days consecutive measurements |
| Chuma 2018 | serum creatinine (SCr) increase of ≥ 0.3 mg/dL, urine volume decrease of 0.5 ml/kg/hr or ≥ 150% from the baseline |
| Fu 2018 | treatment failure: persistence of bacteremia after > 7 days of VCM treatment, or recurrent MRSA infection within 30 days after the first negative blood culture. |
| Huang 2018 | serum creatinine (SCr) increase of ≥ 44.2 µg/L or ≥ 50% from the baseline |
| Mogle 2018 | clinical failure: not meet the clinical success criteria, require other antimicrobial therapy due to persistent bacteremia, or death within 7 days after first therapy |
| Park 2018 | serum creatinine (SCr) increase of ≥ 0.5 mg/dL or ≥ 50% from the baseline SCr for ≥ 2 consecutive measurements |
| de Almeida 2019 | serum creatinine (SCr) increase of ≥ 0.3 mg/dL or ≥ 50% from the baseline within 48 hr |
