# Supplementary figures and images for: The monitoring of vancomycin: a systematic review and meta-analyses of area under the concentration-time curve-guided dosing and trough-guided dosing
Source: BMC Infect Dis. 2021 Feb 6;21:153. doi: 10.1186/s12879-021-05858-6 (PMC7866743; doi:10.1186/s12879-021-05858-6)

## Slide 1
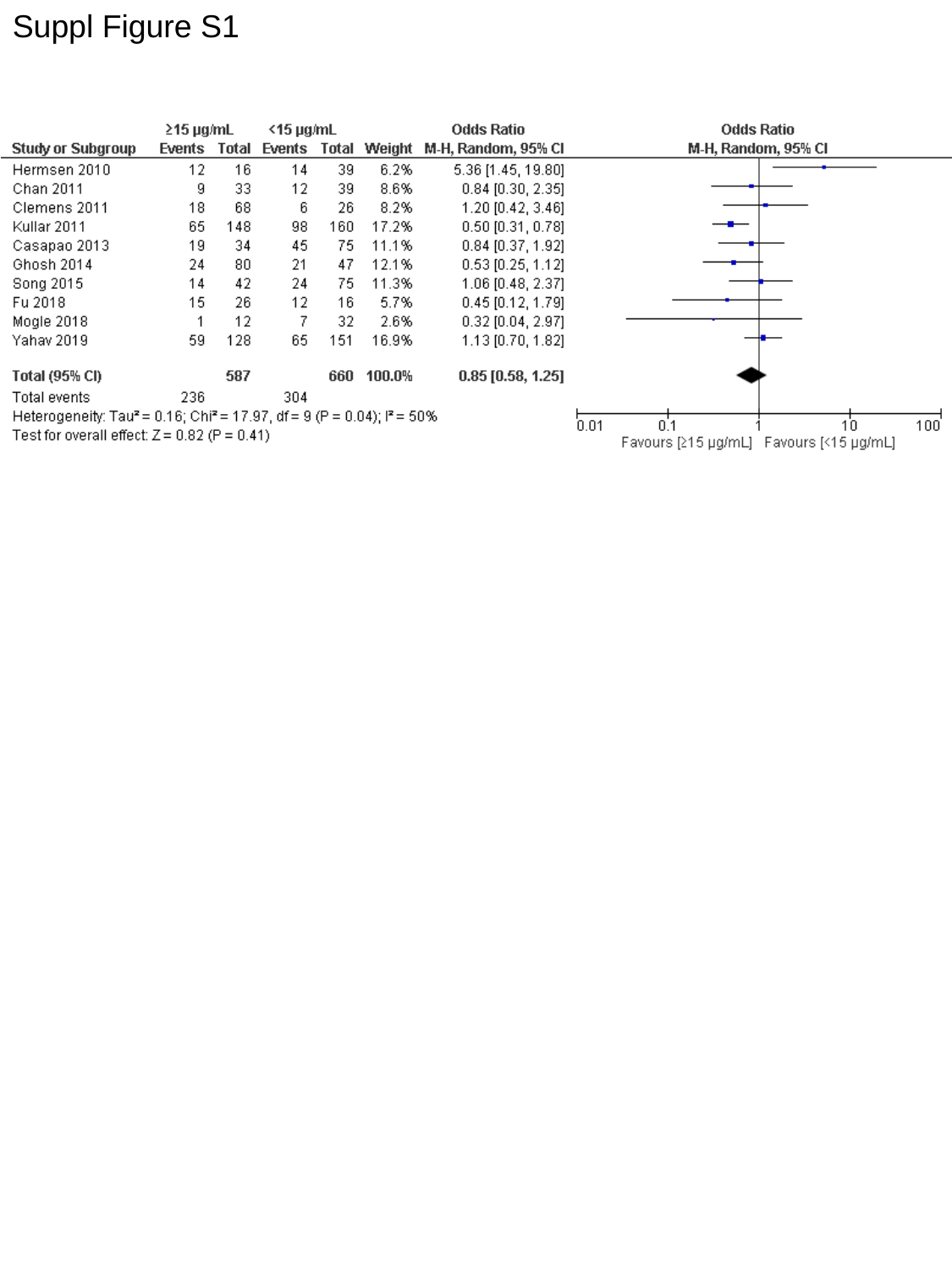

Suppl Figure S1

Supplement: Supplementary file 2 — Additional file 2 : Fig. S1. Forest plot of the treatment failure associated with VCM trough concentra-tion in patients with all MRSA infection. The vertical line indicates no significant difference between the groups compared. Dia-mond shapes and horizontal lines represent ORs and 95% CIs, respectively. Squares in-dicate point estimates, and the size of each square indicates the weight of each study in-cluded in this meta-analysis. VCM trough concentrations were divided into ≥15 μg/mL and < 15 μg/mL. [file 12879_2021_5858_MOESM2_ESM.pptx]
